# Correlation between genotype and phenotype with special attention to hearing in 14 Japanese cases of *NF2*-related schwannomatosis

**DOI:** 10.1038/s41598-023-33812-w

**Published:** 2023-04-22

**Authors:** Naoki Oishi, Masaru Noguchi, Masato Fujioka, Kiyomitsu Nara, Koichiro Wasano, Hideki Mutai, Rie Kawakita, Ryota Tamura, Kosuke Karatsu, Yukina Morimoto, Masahiro Toda, Hiroyuki Ozawa, Tatsuo Matsunaga

**Affiliations:** 1 Department of Otolaryngology-Head and Neck Surgery, Keio University School of Medicine, 35 Shinanomachi, Shinjuku-Ku, Tokyo, 160-8582 Japan; 2 Department of Molecular Genetics, Kitasato University School of Medicine, Kanagawa, Japan; 3 Clinical and Translational Research Center, Keio University Hospital, Tokyo, Japan; 4 Division of Hearing and Balance Research, National Institute of Sensory Organs, National Hospital Organization Tokyo Medical Center, 2-5-1 Higashigaoka, Meguro, Tokyo, 152-8902 Japan; 5 Department of Otolaryngology and Head and Neck Surgery, Tokai University School of Medicine, Kanagawa, Japan; 6 Department of Pediatric Endocrinology and Metabolism, Osaka City General Hospital, Osaka, Japan; 7 Department of Neurosurgery, Keio University School of Medicine, Tokyo, Japan

**Keywords:** Genetics research, Disease genetics, Neurological disorders, Molecular medicine

## Abstract

*NF2*-related schwannomatosis (NF2) is an autosomal dominant genetic disorder caused by variants in the *NF2* gene. Approximately 50% of NF2 patients inherit pathogenic variants, and the remainder acquire de novo variants. NF2 is characterized by development of bilateral vestibular schwannomas. The genetic background of Japanese NF2 cases has not been fully investigated, and the present report performed a genetic analysis of 14 Japanese NF2 cases and examined genotype–phenotype correlations. DNA samples collected from peripheral blood were analyzed by next-generation sequencing, multiplex ligation-dependent probe amplification analysis, and in vitro electrophoresis. Ten cases had pathogenic or likely pathogenic variants in the *NF2* gene, with seven truncating variants and three non-truncating variants. The age of onset in all seven cases with truncating variants was < 20 years. The age of onset significantly differed among cases with truncating *NF2* variants, non-truncating *NF2* variants, and no *NF2* variants. However, the clinical course of tumor growth and hearing deterioration were not predicted only by germline pathogenic *NF2* variants. The rate of truncating variants was higher in the present study than that of previous reports. Genotype–phenotype correlations in the age of onset were present in the analyzed Japanese NF2 cases.

## Introduction

*NF2*-related schwannomatosis (NF2) (formerly Neurofibromatosis type 2) is an autosomal dominant genetic disorder caused by variants in the *NF2* gene located on the long arm of chromosome 22^1–3^. The disease is characterized by development of bilateral vestibular schwannomas (VS) and other benign intracranial and spinal tumors such as meningiomas, schwannomas, and ependymomas^1,2^. The birth incidence rate is approximately 1 in 25,000–33,000, and ~ 50% of patients with non-heritable disease develop de novo variants^1,2,4^. Prevalence is estimated 1 in 50,500–67,700 in UK^4^. The frequencies of tumor presence are 90–95% for bilateral VS, 24–51% for cranial nerve tumors other than VS, 45–58% for intracranial meningiomas, and 63–90% for spinal cord tumors^2^. Histopathological comparison of NF2 VS with sporadic VS revealed that NF2 VS tends to infiltrate nerve fibers and form multicentric patterns^5–7^. In clinical disease course, NF2 VS developed at a younger age, grew faster, and hearing deteriorated more rapidly than in sporadic VS^8,9^. Between 60 and 81% of NF2 cases also develop juvenile cataracts^2^.

In sporadic NF2 cases, *NF2* variants are identified in DNA obtained from peripheral blood in 35–75% of cases^10,11^. The frequency of variants is 51–55% for truncating variants such as nonsense variants and frameshift variants in which protein synthesis is interrupted, 5–9% for non-truncating variants such as missense variants and in-frame deletions in which protein synthesis is not interrupted, and 16–22% for splice site variants^10,11^. Copy number variations (CNVs), such as large deletions, are reported to occur in 17–24% of cases^10,11^. In addition, the overall probable mosaicism rate in de novo NF2 cases is estimated to be approximately 60% using next-generation sequencing^12^.

NF2 exhibits genotype–phenotype correlations^11–19^. Compared with non-truncating and splice site variant cases, truncating variant cases are characterized by younger age of onset, higher risk of death, greater number of tumors other than VS, and lower age at loss of useful hearing^11,13,15–19^. *NF2* variants in the exons 14–15 encoding C-terminal amino acid residues 525–595 show lower risk of cranial meningioma^20^. The severity of NF2 is likely to be milder in mosaic cases than in cases with germline variants^10–14,19^. Previous studies have reported no significant differences between the types of variants in hearing levels or tumor growth rates and sizes during the untreated course^21–26^. In terms of demographic variation, the genetic background of Asian NF2 cases has not been fully studied, and genotype–phenotype correlation in Asian NF2 cases was recently reported in a study^19^. However, there have been no other reports from Asian countries which assessed the relationship between genotype and hearing. Therefore, this report performed a genetic analysis of 14 Japanese NF2 cases and examined the genotype–phenotype correlations between *NF2* gene variants and clinical course, especially hearing prior to intervention.

## Results

Clinical data and detected pathogenic or likely pathogenic (P/LP) variants in each case, variant type, and evaluation of pathogenicity are shown in Table 1. The age for genetic testing was 6–76 years (median 25.5 years). Thirteen of the 14 cases were sporadic, and Case 10 had a first-degree relative with NF2. Case 12 had a unilateral VS with trigeminal schwannoma as well as cataract diagnosed at the age of 42.

**Table 1:** Clinical Manifestations and Genetic Analysis Findings.

Case	Sex	Age of genetic test	VS	Cranial nerve tumor other than VS	Cranial meningioma	Spiral tumor	Cataract	Genotype	Amino acid change	Type of variant	Pathogenicity	Reference
1	M	30	B	+	+	+		c.[586C > T];[ =]	p.[Arg196*];[ =]	Nonsense	Pathogenic	^27^
2	F	22	B	+		+		c.[1515_1532delinsT];[ =]	p.[Ser506Tyrfs*2];[ =]	Frameshift	Pathogenic	This study
3	M	23	B	+	+	+		c.[586C > T];[ =]	p.[Arg196*];[ =]	Nonsense	Pathogenic	^27^
4	M	17	B	+	+	+	+	c.[407_423dup];[ =]	p.[Ala142Argfs*38];[ =]	Frameshift	Pathogenic	This study
5	M	24	B	+	+	+		c.[1408C > T];[ =]	p.[Gln470*];[ =]	Nonsense	Pathogenic	^13^
6	M	6	B	+	+			c.[784C > T];[ =]	p.[Arg262*];[ =]	Nonsense	Pathogenic	^28^
7	F	24	B		+	+	+	c.[552G > A];[ =]	p.[Trp184*];[ =]	Nonsense	Pathogenic	^29^
8	F	45	B	+	+			c.[516 + 1G > A];[ =]		Splice site	Likely pathogenic	^30^
9	F	41	B	+	+	+		c.[357_359del];[ =]	p.[Phe119del];[ =]	In-frame deletion	Likely pathogenic	^31^
10	M	27	B					c.[(990_1102)_(1102_1182)del];[ =]		Exon del	Likely pathogenic	This study
11	F	47	B	+	+	+		Not detected	Not detected	Not detected	Not detected	
12	M	52	U	+			+	Not detected	Not detected	Not detected	Not detected	
13	M	19	B	+				Not detected	Not detected	Not detected	Not detected	
14	M	76	B					Not detected	Not detected	Not detected	Not detected	

*M* Male, *F* Female, *VS* Vestibular Schwannoma, *B* Bilateral, *U* Unilateral, + : presence of NF2-related lesion, Reference: The referenced reports represent prior identification of the same variant in an unrelated individual, Reference Sequence ID: LRG_511t1 (NM_000268.4), Genomic Coordinate of variants: Case1 and 3: NC_000022.10:g.30051652C > T, Case2: NC_000022.10:g.30074253_30074270delinsT, Case4: NC_000022.10:g.30038234_30038250dup, Case5: NC_000022.10:g.30070892C > T, Case6: NC_000022.10:g.30057302C > T, Case7: NC_000022.10:g.30051618G > A, Case8: NC_000022.10:g.30050715G > A, Case9: NC_000022.10:g.30035195_30035197del, Case10: NC_000022.10:g.(30064426_30067917)_(30067917_ 30069317)del.

**Figure 1:**
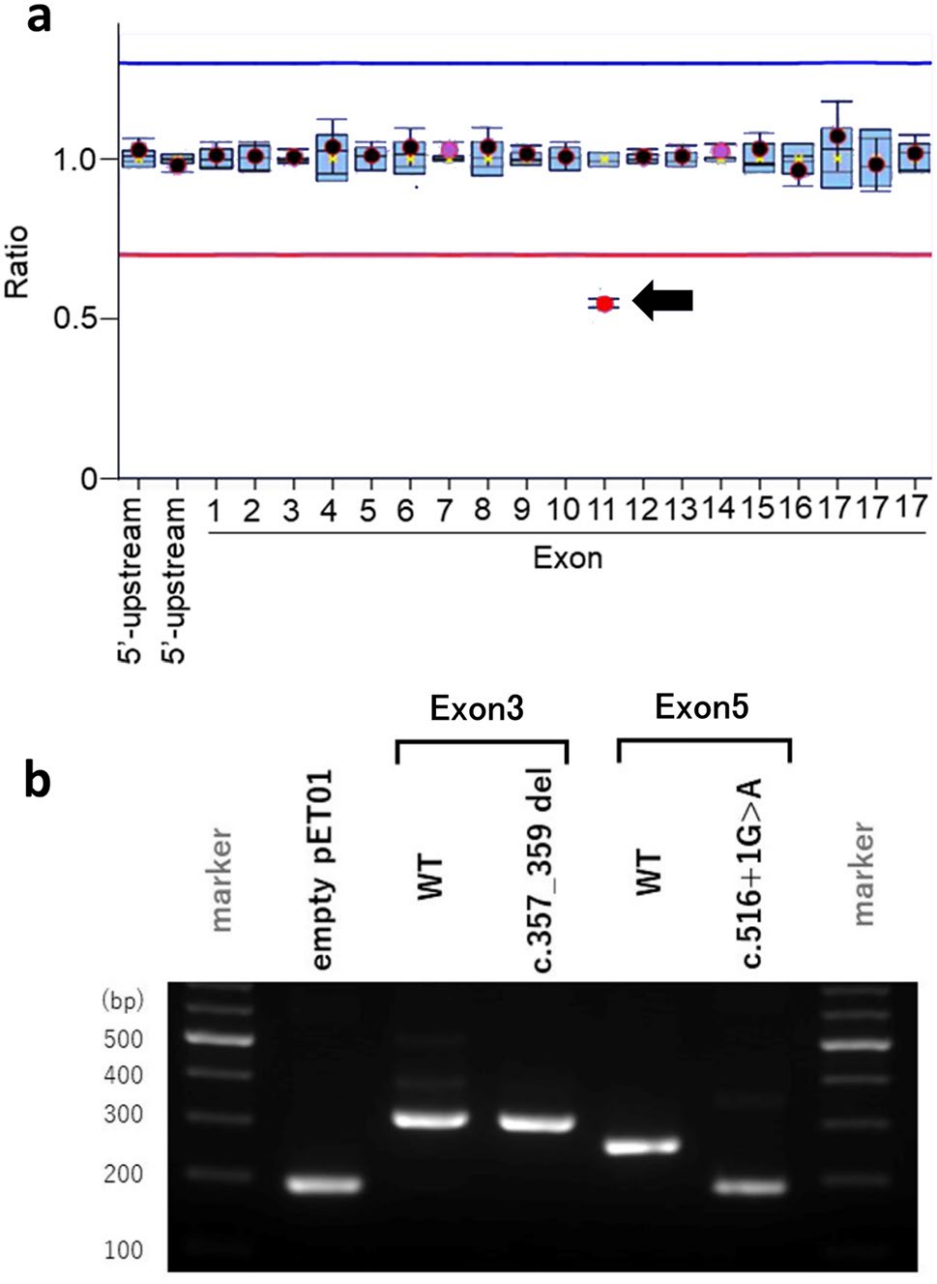
Genetic analysis of variants in addition to sequencing. **(a)** Detection of exon deletions in *NF2* by multiplex ligation-dependent probe amplification (MLPA). The ratio of exon 11 was approximately 0.5 in Case 10 (arrow), suggesting that the exon was deleted in a heterozygous manner. The ratio for normalized fluorescence intensity is represented on the Y-axis. MLPA probes for the indicated exons are shown on the X-axis. *Circles* indicate the ratios for experimental samples and *boxes* indicate the ratios for the normal controls (n = 3). *Error bars* indicate estimated 95% confidence intervals in the samples. *Blue* and *red* lines represent the upper and lower limits for normal range. **(b)** Evaluation of splicing in variants detected in Case 8 and Case 9. In Case 9 (c.357_359del), the band was observed in the same position as wild type (WT). In Case 8 (c.516 + 1G > A), the band was observed in the same position as the empty vector (empty pET01), in which exon 5 (69 bp) was predicted to be skipped by splicing.

Targeted resequencing analysis revealed truncating variants in seven cases (Cases 1–7). Five of the cases with truncating variants had nonsense variants, and two had frameshift variants. In addition, one case (Case 8) had a splice site variant and another case (Case 9) had an in-frame deletion. In five cases, (Cases 10–14), in which P/LP variants were not detected by targeted resequencing analysis, multiplex ligation-dependent probe amplification (MLPA) analysis was performed. Heterozygous deletion of exon 11 was detected in Case 10 (Fig. 1a) and no CNVs were detected in the other four cases. Due to existence of a polymorphic variant, c.1113C > T, on the sequence corresponding to the MLPA probe in case 10, we further confirmed the deletion by a read-depth analysis of the targeted-sequencing data (Supplementary Figure S1). Because exon 11 is 123 bp, deletion of the exon was considered to result in production of in-frame non-truncating NF2 protein. The variant was predicted to remove less than 10% of the protein and evaluated as likely pathogenic.

For Case 8, which had a splice variant, and Case 9, which had an in-frame deletion, the effects of the variants on protein splicing were tested by in vitro electrophoresis. In Case 8, the variant was detected at the donor site of exon 5 and predicted as a splice site variant. A band was detected at the same position as the empty vector, and exon 5 (69 bp) was predicted to be skipped (Fig. 1b, Supplementary Figure S2). In Case 9, the deletion was detected at exon 3 and predicted as an in-frame deletion. The deletion did not have an identifiable splicing effect (Fig. 1b, Supplementary Figure S2). Therefore, both Cases 8 and 9 were predicted to have non-truncating and in-frame deletions. Both variants were predicted to remove less than 10% of the protein and evaluated as likely pathogenic.

**Figure 2:**
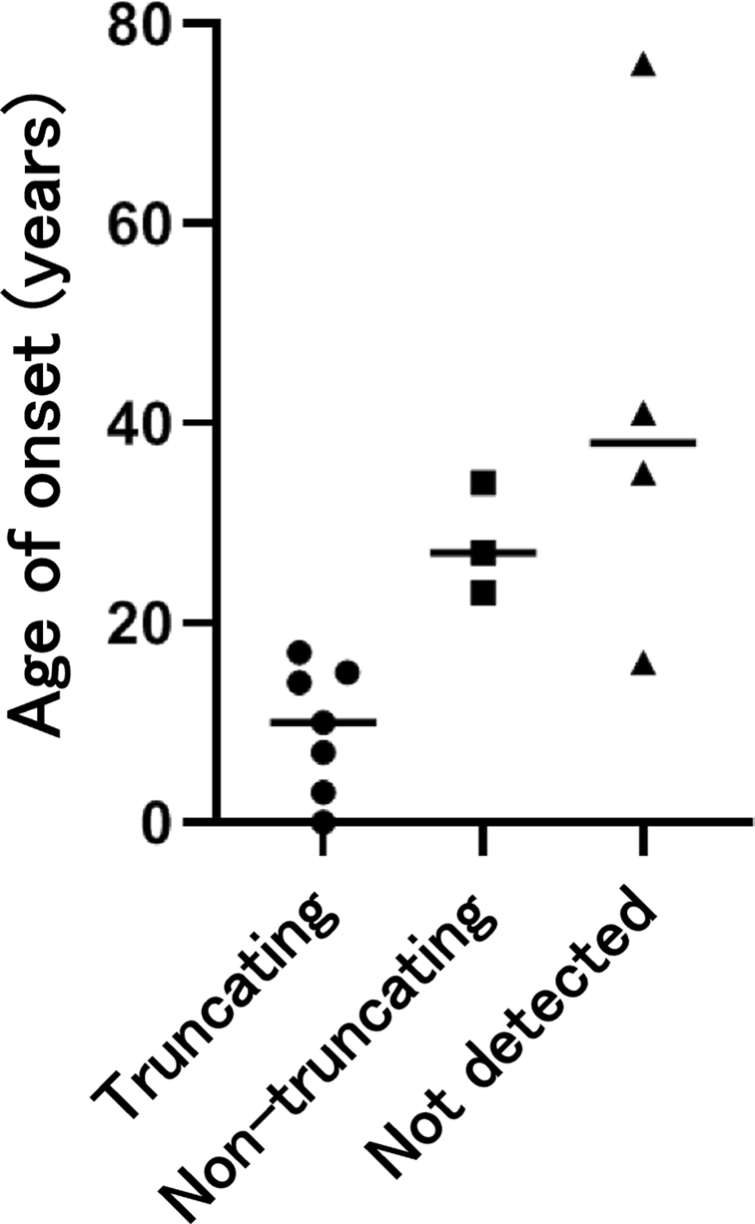
Variant type and age of onset. The difference in age of onset among the three groups was statistically significant (*p *= 0.0014, Kruskal–Wallis test). All cases of truncating variants developed NF2 prior to the age of 20 years. The median age of onset of truncating variant cases was significantly lower than that of the ‘not detected’ cases (*p *= 0.0151).

The age of onset of NF2-related symptoms is shown in Fig. 2. The ages of onset of all seven cases predicted to have truncating variants were < 20 years (median 10 years (0–17 years)). The median age of onset in the three cases predicted to have non-truncating variants was 27 years (23–34 years), and the median age of onset of the other four cases with no P/LP variants or CNV detected was 38 years (16–76 years). The difference of age of onset among the three groups was statistically significant (*p *= 0.0014). A Dunn’s multiple comparisons test revealed that the median age of onset of truncating variant cases was significantly lower than that of the ‘not detected’ cases (*p *= 0.0151).

**Figure 3:**
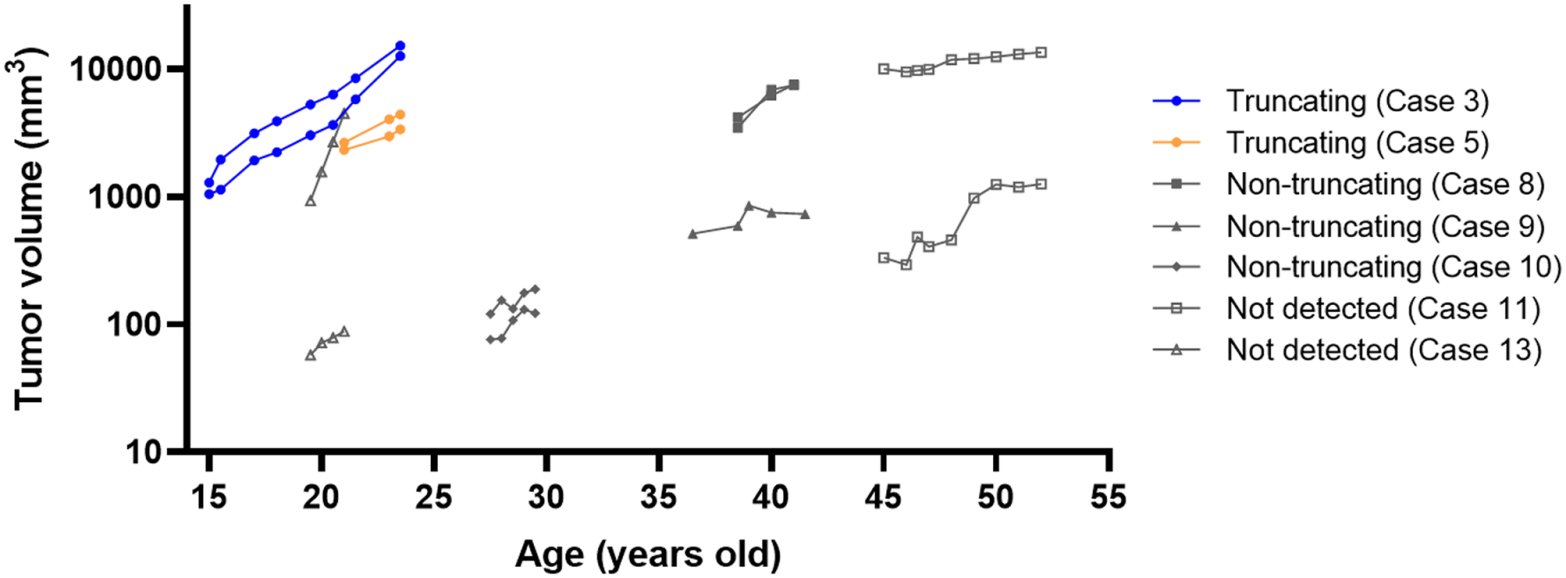
Variant types and natural courses of tumor volume. VS volumes of 13 tumors in seven cases were examined in the natural disease courses without any interventions. Tumor volumes increased in most of the affected ears. The tumor growth rates varied among affected ears, and no consistent trend was detected between variant types. The growth rates were asymmetric even within the same case.

Tumor volumes of VS were followed for more than one year in 13 tumors of seven cases (Cases 3, 5, 8, 9, 10, 11, 13) (Fig. 3). The average follow-up period was 4.2 ± 2.6 years (median 3.0 years). Tumor volumes increased during follow-up in most of the affected ears. The average growth rate was 1067.5 ± 621.5 mm^3^/year in the four truncating variant ears and 674.8 ± 905.9 mm^3^/year in the other nine ears. The tumor growth rate varied among the affected ears, and no consistent trend was detected between variant types. The courses of both ears were followed in six cases, and tumor volumes had asymmetric changes in two out of the six cases (Cases 11, and 13).

**Figure 4:**
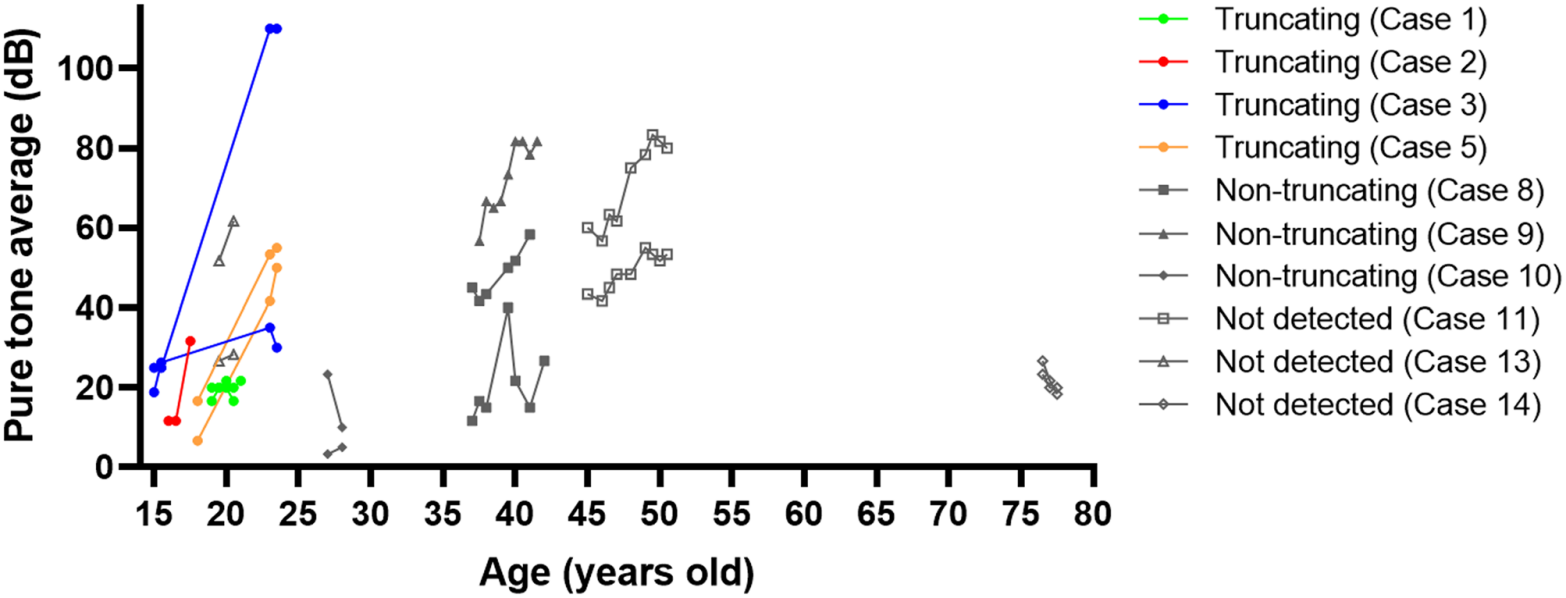
Variant type and natural course of hearing level. Pure-tone averages (PTAs) threshold of 18 ears in ten cases were examined in disease courses without interventions. PTAs tended to deteriorate overall. The increase rates of PTAs varied among affected ears, and no consistent trends were detected between variant types. PTA changes were asymmetric even within the same cases.

Pure-tone averages (PTAs) were followed for > 1 year in 18 ears of ten cases (Cases 1, 2, 3, 5, 8, 9, 10, 11, 13, 14) (Fig. 4). The average follow-up period was 3.5 ± 2.6 years (median 3 years). PTAs increased in most of the affected ears. The average increase rate was 6.0 ± 5.3 dB/year in seven ears of truncating variant cases and 0.3 ± 6.4 dB/year in the remaining eleven ears. The PTA increase rates varied among affected ears, and no consistent trend was detected between variant types. The courses of both ears were followed in eight cases, with asymmetric PTA changes in six of the eight cases (Cases 1, 3, 8, 10, 11, and 13).

**Figure 5:**
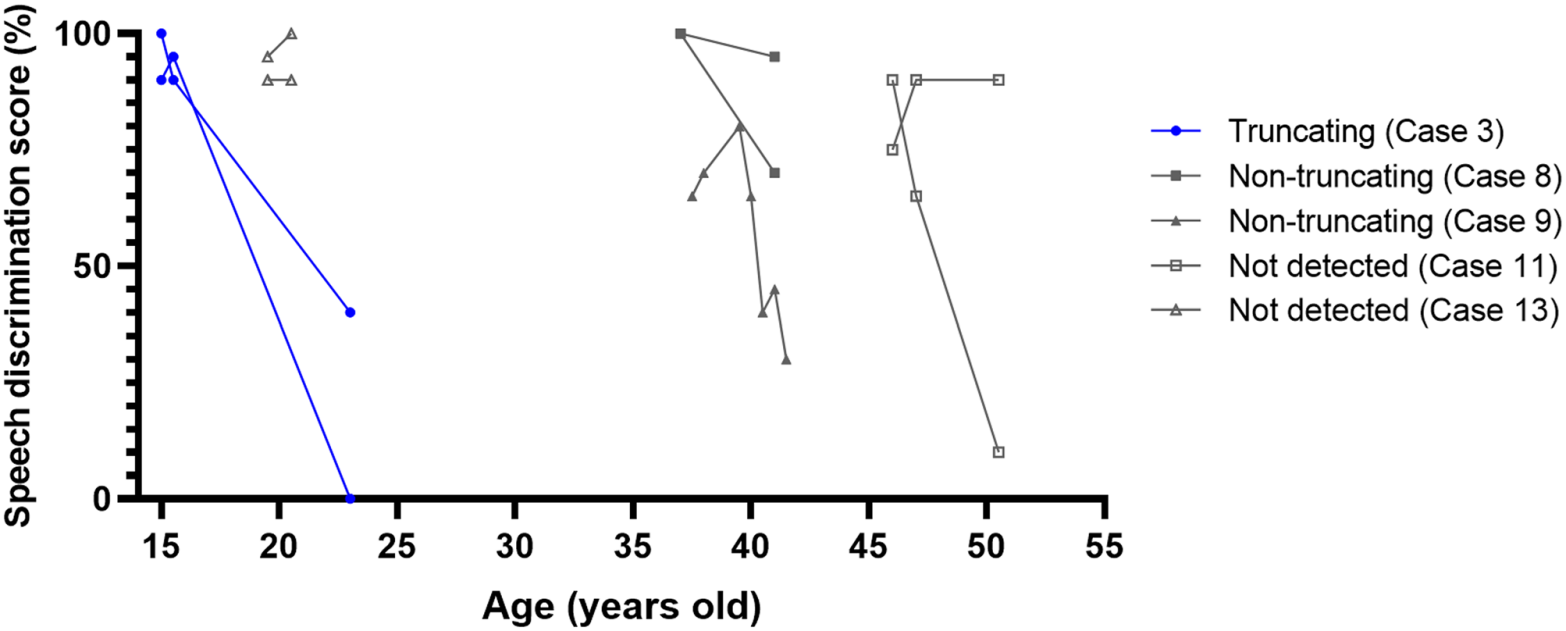
Variant types and natural course of highest speech discrimination scores. Speech discrimination scores (SDSs) of nine ears in five cases were examined in the natural disease course without any interventions. SDSs tended to decline overall. The SDS deterioration rate varied among affected ears, and no consistent trend was detected between variant types. SDS changes were asymmetric even within the same case.

Speech discrimination scores (SDSs) were followed for > 1 year in nine ears of five cases (Cases 3, 8, 9, 11, 13) (Fig. 5). The average follow-up period was 4.3 ± 2.5 years (median 4.0 years). SDSs deteriorated in most of the affected ears. The average deterioration rate was 9.4 ± 2.7%/year in two ears with the truncating variant and 3.8 ± 9.0%/year in the other seven ears. The SDS deterioration rate varied among affected ears, but no consistent trend was detected among variant types. The courses of both ears were followed in four cases, revealing asymmetric SDS changes in three of the four cases (Cases 8, 11, and 13).

## Discussion

In the 14 evaluated Japanese NF2 cases, P/LP variants were detected in ten cases (71.4%). The variant detection rate in peripheral blood was consistent with that of previous reports (35–75%)^10,11^. None of the 14 patients had a family history of NF2, which is consisted with the results of Teranishi et al.^19^, indicating the low inheritance of NF2 in Japanese. In the de novo NF2 cases, mosaicism, which was not evaluated in this study, was likely to be present considering the high estimated rate of mosaicism (60%)^12^. Seven of the ten cases with detected P/LP variants (70%) had truncating variants. Previously reported rates of truncating variants in cases with pathogenic variants were approximately 50% in Western countries^10,11^, as well as in Japanese subjects^14^. Prior studies have not identified differences in NF2 morbidity between races and ethnic groups^32,33^. Thus, the rate of truncating variants detected in the present study could have been higher because > 50% of the participants were younger patients (< 20 years). All truncating variant cases developed NF2 at < 20 years, and the age of onset was significantly lower than in cases with no detected variants, as in previous reports^11–13,15,16^. Other than these correlations, genotype–phenotype correlation was not identified for tumor growth, hearing levels, and speech discrimination scores in contrast to the previous reports^11,19^. This might be explained by the small number of the patients, limited follow up, and lack of mosaicism determination which are the limitations of the present study.

The increased rate of tumor growth during the untreated period varied among affected ears, and no consistent trend was detected between variant types. The courses of tumor growth progression varied bilaterally even within the same case. Therefore, to predict tumor growth rate, it is necessary to evaluate factors other than germline variants of the *NF2* gene from peripheral blood. As the candidates of such predictive factors, tumor growth rate could also be affected by variants in other alleles (second hit) in individual tumors^34,35^. Further, in reports of sporadic VS, increases in tumor sizes were associated with expression of vascular endothelial growth factor (VEGF) and its receptors^36,37^.

No consistent trend in hearing deterioration was detected between variant types. No significant differences in hearing level have been reported between variant types, nor have correlations between hearing level and tumor size^21–24^. One potential mechanism of hearing deterioration in VS is tumor secretory factors such as TNF alpha^38,39^. To predict the deterioration of hearing, it could be necessary to consider factors other than the *NF2* gene, including the anatomical positional relationship between the tumor and the nerves, the degree of impaired blood flow to the cochlear nerve and cochlea, and the influence of tumor secretory factors. Halliday et al. and Teranishi et al. reported genetic severity scores based on the type of variant, respectively^9,19^. When the cases in which pathogenic variants were detected in this report were categorized based on the two different scoring systems, the results were identical in Cases 8 and 10. However, Case 9 could not be evaluated based on the Teranishi scoring system because the system has no corresponding score to in-frame deletion.

In four cases (Cases 11, 12, 13, and 14), P/LP variants were not detected, nor was CNV. If no P/LP variants are detected in the peripheral blood, these cases could be mosaic, and the clinical courses could be milder with a later age of onset^10–13^. In the present study, the age of onset in Cases 11 and 12 were both middle-aged, and that of Case 14 was > 70 years, while the age of onset of Case 13 was 17 years old. To determine if these cases were mosaic, it would be necessary to collect samples such as in tumors rather than peripheral blood and investigate whether P/LP variants are detected^14^. It is also possible that no P/LP variant was detected due to chromosomal structural abnormalities such as ring chromosome which could not be detected by the genetic analysis used. To detect these changes, microarray analysis would be effective. These analyses in a larger number of patients would determine genetic characteristics and genotype–phenotype correlations of NF2 occurring in Asian patients.

## Conclusion

In the 14 Japanese NF2 cases evaluated in the study, P/LP variants were detected in ten cases (71.4%), and seven of the ten cases (70%) were truncating variants. Genotype–phenotype correlations in the age of onset were detected. However, it would be necessary to consider factors in addition to *NF2* germline P/LP variants to predict phenotypic characteristics such as tumor growth and hearing deterioration.

## Materials and methods

### Participants

Fourteen cases diagnosed with NF2 using Manchester criteria^40^ at Keio University Hospital, National Hospital Organization Tokyo Medical Center, and Osaka City General Hospital, from September 2016 to August 2020, were enrolled in the study. Nine cases were male, and five were female. Written informed consent was obtained from all cases. The study was approved by the ethics committees of Keio University School of Medicine, National Hospital Organization Tokyo Medical Center, and Osaka City General Hospital (approval numbers 20150235, R20-184, and 1601110, respectively). The study was conducted in accordance with the principles of the Declaration of Helsinki.

### DNA sequencing

DNAs were extracted from peripheral blood leukocytes using Genomix (Biologica, Nagoya, Japan). Targeted resequencing, including exonic regions of *NF2*, was performed using the SureSelect target enrichment system (Agilent Technologies, CA, USA) or NEXTFLEX Rapid XP DNA seq kit (Perkin-Elmer, MA, USA), followed by target resequencing with a next-generation sequencer (NextSeq platform, Illumina, CA, USA). Variant calling was conducted with SureCall (Agilent Technologies)^41^. The details are described in our previous report^42^.

Candidate variants from panel analysis were verified by Sanger sequencing using a standard protocol. PCR reactions were conducted to amplify the corresponding exons using PrimeSTAR DNA polymerase (TAKARA BIO, Kyoto, Japan) with the primers listed in the Supplementary Table S1. The pathogenicity of the detected variants was evaluated according to the American College of Medical Genetics and Genomics (ACMG) guidelines^43^. For loss of function variants, we also used the recommendation by the ClinGen sequence variant interpretation working group^44^. Subjects with no candidate variant were further analyzed by the MLPA method to assess CNVs using SALSA MLPA *NF2* probemix (MRC Holland, Amsterdam, The Netherlands) according to manufacturer’s instructions. MLPA results were confirmed with read-depth analysis using DepthOfCoverage version 4.1.9.0 in gatk4^45^.

### Assessment of splicing

The effects of splicing were evaluated for cases predicted to have non-truncating pathogenic variants. Exons and introns containing wild-type and predicted pathogenic variants were introduced into the pET01 vector (MoBiTec, Göttingen, Germany) cleaved by the XhoI (R0146S, NEB). After transduction of the vectors into *Escherichia coli*, introduction of the target sequences was confirmed. HEK293T cells were transfected with empty vectors, wild-type vectors, and vectors containing the predicted pathogenic variants by Effectene (301425, Qiagen). Moreover, after culturing HEK293T cells for 40–48 h, RNAs were extracted using the Quick-RNA MiniPrep Plus kit (R1057, Zymo Research). Reverse transcriptions of the obtained RNAs were performed using SuperScript III Reverse Transcriptase (18080044, Thermo Fisher Scientific) to prepare cDNAs. After cDNAs were amplified by PCR using Phusion High-Fidelity DNA polymerase (M0530, NEB), product length was confirmed by electrophoresis. The PCR primers were 5'-CCTGGCTGCCCAGGCTTTTGTCAACA-3' and 5'-CCACCTCCAGTGCCAAGGTCTGAAGGTCA-3'.

### Clinical characteristics

The relationship between variant type and clinical information such as NF2-related lesions, age of onset, VS tumor volume calculated from MRI, PTA, and highest SDS was evaluated prior to intervention. Clinical information was obtained retrospectively from medical records. PTA refers to the average hearing threshold at 0.5, 1, and 2 kHz. SDS refers to the percentage of words correctly recognized and repeated. The clinical courses of tumor volume, PTA, and SDS were evaluated for cases followed more than 1 year before therapeutic interventions for VS. Regarding the age of NF2-related symptom onset, the truncating variant group, non-truncating variant group, and ‘not detected’ group were compared using a Kruskal–Wallis test. *p-*values < 0.05 were considered statistically significant. IBM SPSS Statistics version 25 (IBM, NY, USA) was used for statistical analysis.

## Supplementary Information

Supplementary Information.

## Data Availability

All the *NF2* variants detected in this study and clinical features of subjects are included within this article and deposited to Medical Genomics Japan Variant Database (MGeND, https://mgend.med.kyoto-u.ac.jp/ , accession number: MGS000062). The genomic data supporting the findings of this study may be made available to qualified investigators upon request with appropriate institutional review board approval and execution of a data use agreement with the corresponding authors.
