# Supplementary material for: Correlation between genotype and phenotype with special attention to hearing in 14 Japanese cases of NF2-related schwannomatosis
Source: Sci Rep. 2023 Apr 22;13:6595. doi: 10.1038/s41598-023-33812-w (PMC10122645; doi:10.1038/s41598-023-33812-w)
Supplement: Supplementary file 1 — Supplementary Information. [file 41598_2023_33812_MOESM1_ESM.pdf]

Supplementary information

Correlation between genotype and phenotype with special attention to hearing in 14 Japanese cases of NF2

Naoki Oishi, Masaru Noguchi, Masato Fujioka, Kiyomitsu Nara, Koichiro Wasano, Hideki Mutai, Rie Kawakita, Ryota Tamura, Kosuke Karatsu, Yukina Morimoto, Masahiro Toda, Hiroyuki Ozawa, Tatsuo Matsunaga

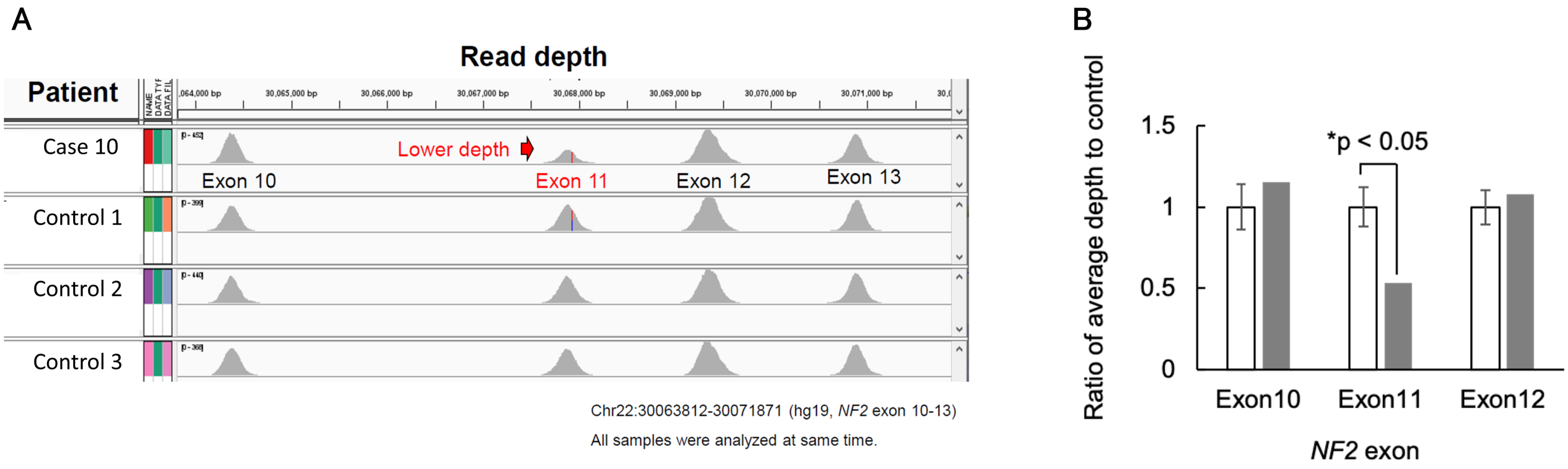

Figure S1 Read depth analysis for exon-11 deletion detected in Case 10

A: The read depth for exon 10-13 are shown. **B**: The ratio of average depth to controls for exon 10-12 are shown. The values were normalized by dividing by the average of the five controls' average depths. *Open bars*, average of controls (n = 5); *solid bars*, Case 10. Note that the value of exon 11 in the case is about half, which indicates that the exon of an allele is deleted. Experiments including library preparation and sequencing were done simultaneously for all samples, including controls. Significance in the difference between the two groups was tested using a Student *t*-test.

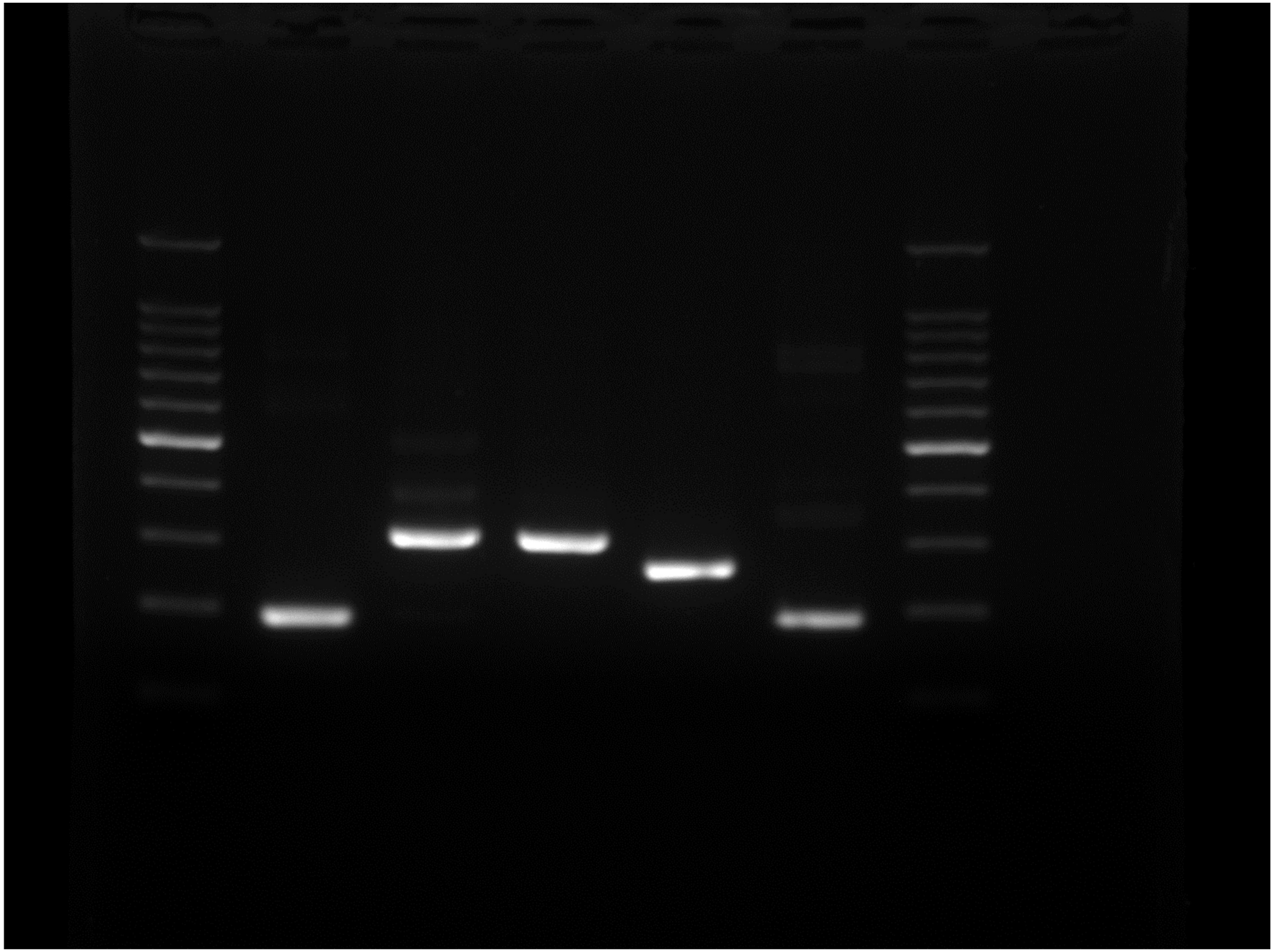

Figure S2 Original image of Figure 1b

Supplementary Table S1: Primers used to confirm variants detected by NGS

Case	**Forward primer**[#1,2]	**Reverse primer**[#1,2]
1	*NF2*_ex6_F1: tgtaaaacgacggccagtGTGCCTTATTTACACGCCCTC	*NF2*_ex6_R1: caggaaacagctatgaccTGAAGACCAACGTTACTCCC
2	*NF2*_ex14_F1: tgtaaaacgacggccagtCTGCTGGAGGATCGGTTGTC	*NF2*_ex14_R1: caggaaacagctatgaccGCAGAGCAAACCTTTGCAGG
3[#3]	*NF2*_ex6_F2: tgtaaaacgacggccagtGGTCTCTGTGATGTATCGCCC	*NF2*_ex6_R2: caggaaacagctatgaccACACACAAAACTGAAGACCAACG
4	*NF2*_ex4_F1: tgtaaaacgacggccagtCTCATTAGAACGCCGTGAGG	*NF2*_ex4_R1: caggaaacagctatgaccAACATCTCACTTTCTGGGGCAA
5	*NF2*_ex13_F1: tgtaaaacgacggccagtTCACCTCTTTGGGTGCCATC	*NF2*_ex13_R1: caggaaacagctatgaccTCCCTAACTGCAGGCTCTCT
6	*NF2*_ex8_F1: tgtaaaacgacggccagtCCAGTGACAGAAGCCCTCAG	*NF2*_ex8_R1: caggaaacagctatgaccCACCCTCAAAGCCTGGGAAT
7	*NF2*_ex6_F2: tgtaaaacgacggccagtGGTCTCTGTGATGTATCGCCC	*NF2*_ex6_R2: caggaaacagctatgaccACACACAAAACTGAAGACCAACG
8	*NF2*_ex5_F1: tgtaaaacgacggccagtGGAGCTGGGAGGGAATGAGA	*NF2*_ex5_R1: caggaaacagctatgaccCATGCTAGTCCTGGTGACCC
9	*NF2*_ex3_F1: tgtaaaacgacggccagtTGAGGGTAGCACAGGAGGAA	*NF2*_ex3_R1: caggaaacagctatgaccCTCTGAGGCCAACTCTGCAA

[#1]Lowercases indicate sequences of M13 sequencing primers. [#2]Annealing temperature was 63℃ for all primers.

[#3]Different primers were used even to amplify same exon because the sample had a polymorphism at the primer sequence.
